# Genetic diversity and differentiation of populations of *Anthyllis vulneraria* along elevational and latitudinal gradients

**DOI:** 10.1002/ece3.9167

**Published:** 2022-08-04

**Authors:** Laura Daco, Diethart Matthies, Sylvie Hermant, Guy Colling

**Affiliations:** ^1^ Musée national d'histoire naturelle Luxembourg Luxembourg; ^2^ University of Marburg, Department of Biology Marburg Germany; ^3^ Fondation faune‐flore Luxembourg Luxembourg

**Keywords:** abundant centre model, climate change, founder effect, isolation by distance, Pleistocene glaciation, population size effect

## Abstract

The abundant centre model (ACM) predicts that the suitability of environmental conditions for a species decreases from the centre of its distribution toward its range periphery and, consequently, its populations will become scarcer, smaller and more isolated, resulting in lower genetic diversity and increased differentiation. However, little is known about whether genetic diversity shows similar patterns along elevational and latitudinal gradients with similar changes in important environmental conditions. Using microsatellite markers, we studied the genetic diversity and structure of 20 populations each of *Anthyllis vulneraria* along elevational gradients in the Alps from the valleys to the elevational limit (2500 m) and along a latitudinal gradient (2500 km) from Central Europe to the range margin in northern Scandinavia. Both types of gradients corresponded to an 11.5°C difference in mean annual temperature. Genetic diversity strongly declined and differentiation increased with latitude in line with the predictions of the ACM. However, as population size did not decline with latitude and genetic diversity was not related to population size in *A. vulneraria*, this pattern is not likely to be due to less favorable conditions in the North, but due to serial founder effects during the post‐glacial recolonization process. Genetic diversity was not related to elevation, but we found significant isolation by distance along both gradients, although the elevational gradient was shorter by orders of magnitude. Subarctic populations differed genetically from alpine populations indicating that the northern populations did not originate from high elevational Alpine ones. Our results support the notion that postglacial latitudinal colonization over large distances resulted in a larger loss of genetic diversity than elevational range shifts. The lack of genetic diversity in subarctic populations may threaten their long‐term persistence in the face of climate change, whereas alpine populations could benefit from gene flow from low‐elevation populations.

## Graphical abstract

We studied the genetic diversity and population structure of 40 populations of the widespread plant species *Anthyllis vulneraria* (kidney vetch) along an elevational gradient in the Alps and a latitudinal gradient from Central Europe to northern Scandinavia. Genetic diversity strongly decreased and genetic differentiation among populations increased with latitude, suggesting that founder effects played an important role in the establishment of high‐latitude populations during the post‐glacial recolonization of formerly glaciated areas. Our results also indicate that subarctic populations differed genetically from alpine populations suggesting that the northern populations did not originate from high elevational Alpine ones.

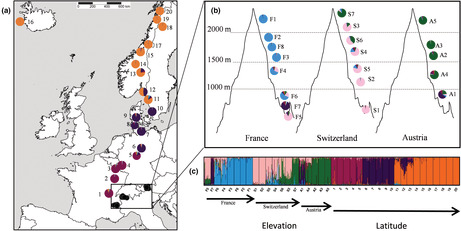

## 1 INTRODUCTION

The genetic diversity of populations and their differentiation is influenced both by contemporary evolutionary processes like gene flow, genetic drift, and natural selection and by the history of a species (Frankham et al., 2017). In small and isolated populations genetic variation is often strongly reduced and genetic divergence among populations increased because of reduced gene flow and stronger genetic drift (Aguilar et al., 2008; Fischer & Matthies, 1998; Schlaepfer et al., 2018). Bottlenecks and founder effects may also have strong negative effects on genetic diversity (Frankham et al., 2017). Due to environmental gradients, the processes that influence genetic diversity often vary across the distributional range of a species. The abundant centre model (ACM) predicts that the suitability of environmental conditions for a species decreases from the centre of its distribution to its range periphery and consequently its populations will become scarcer, smaller and more isolated toward the range limits (Brown, 1984; Sagarin & Gaines, 2002). Genetic consequences of the decrease in the number and size of populations toward the range periphery and their increasing isolation are predicted to be reduced genetic diversity within populations and increased genetic differentiation among populations due to increased genetic drift and reduced gene flow at the periphery of the distribution of a species (Eckert et al., 2008; Hardie & Hutchings, 2010; López‐Delgado & Meirmans, 2022; Sexton et al., 2009). However, while a recent review found that only approximately half of the available studies supported these predictions (Pironon et al., 2017), a study of 91 North American native plants found strong support for the ACM (López‐Delgado & Meirmans, 2022).

The genetic diversity and population structure of plant species across its range may also be influenced by range shifts linked to Pleistocene climate oscillations (Harter et al., 2015), which had a major impact on the present distribution of plants (Hewitt, 2000). Populations typically retained high levels of genetic diversity and allelic richness in the glacial refugia where they survived during the ice ages (Beatty & Provan, 2011; López‐Delgado & Meirmans, 2022). With the retreat of the ice shields after climate warming, individuals from the surviving populations colonized the new suitable habitats. Postglacial colonization over long distances by serial founder events often resulted in a decline in genetic diversity. Thus, populations often have less genetic variation at higher latitudes and are genetically more differentiated than at lower latitudes (Ehrich et al., 2007; Hewitt, 2004; López‐Delgado & Meirmans, 2022). However, northern populations in Europe may also have become established by a massive migration of cold‐tolerant plants from the Central European tundra into the forelands of retreating ice shields during the window of opportunity before tree species migrated North. In this scenario one would expect that genetic diversity within populations would not decrease toward the northern periphery. An example is the artic‐alpine species *Dryas octapetala* whose genetic diversity in Scandinavian populations is high and today's arctic populations of the European cluster are closely related to alpine populations indicating a common origin in the tundra south of the Scandinavian ice‐shield (Skrede et al., 2006).

Important environmental conditions (e.g. temperature) that influence the suitability of habitats for a species may change along elevational gradients in similar ways as with latitude and influence the balance between drift and gene flow. However, there are also important differences between the changes in environmental conditions along the two types of gradients, including those in day length, irradiance, CO_2_ partial pressure, and precipitation (Körner, 2007). Moreover, elevational gradients are much shorter than latitudinal ones, and thus gene flow between populations is more likely (Hahn et al., 2012; Halbritter et al., 2015). Four patterns of genetic diversity along elevational gradients have been found (Itino & Hirao, 2016; Ohsawa & Ide, 2008): (1) Populations at intermediate elevations have higher genetic diversity than populations at both higher and lower elevations because conditions at intermediate (i.e. “central”) elevations are optimal, whereas populations at the lower and upper elevation edges are more affected by restricted gene flow, genetic drift and founder effects, leading to reduced genetic diversity (Byars et al., 2009; Herrera & Bazaga, 2008; Meng et al., 2019; Ohsawa et al., 2007). (2) Populations at low elevations are genetically most diverse (Premoli, 2003; Quiroga & Premoli, 2007) because conditions at low elevations are best and founder effects have occurred during upward range expansion. (3) Genetic diversity increases with elevation in species whose main habitats are in the alpine zone, or whose populations are negatively impacted by human activities at lower elevations (e.g. Halbritter et al., 2015; Reisch et al., 2005; Shi et al., 2011). Finally, genetic diversity may be unrelated to elevation due to extensive gene flow or random variation caused by strong local factors (Hahn et al., 2012; Halbritter et al., 2015). Reviews of studies on genetic diversity along elevational gradients have found no general patterns (Itino & Hirao, 2016; Ohsawa & Ide, 2008), and little is known about whether latitudinal and elevational gradients in environmental conditions have similar effects on the genetic structure and diversity of a plant species. A better understanding of patterns of genetic diversity and differentiation along these gradients is important because genetic diversity will determine the potential of populations to adapt to ongoing global change (Jump et al., 2009).

The effects of the two types of gradients should ideally be compared in species that have both a large latitudinal and elevation extension (Halbritter et al., 2015). We chose the kidney vetch *Anthyllis vulneraria* (Fabaceae) as a model species to study the patterns of genetic variability and differentiation as influenced by latitude and elevation because it has an exceptionally wide geographic and elevational distribution in Europe. The large distribution of *A. vulneraria* allowed us to study general genetic patterns that cannot be detected in arctic‐alpine species or in rare species with fragmented and isolated populations. We studied the genetic diversity and structure of *A. vulneraria* along two gradients chosen to correspond to a change of 11.5°C in annual mean temperature: a latitudinal gradient of c. 2400 km from Central Europe to Iceland and northern Norway and three elevational gradients of c. 2000 m elevational difference in the European Alps (see also Daco et al., 2021). The latitudinal gradient ranged from the centre of the distribution of *A. vulneraria* to its northern range limit and the elevational gradient in the Alps from the valleys to the upper elevational edge of its distribution. We address the following specific questions: (1) Does the genetic diversity of *A. vulneraria* vary similarly along gradients of elevation and latitude? (2) Are patterns of genetic differentiation similar along the two types of gradients?

## 2 MATERIALS AND METHODS

### 2.1 Study species

*Anthyllis vulneraria* L. (Fabaceae) is a diploid biennial to perennial herb of nutrient‐poor calcareous grasslands and screes. Its distribution is exceptionally wide as it occurs from the North of the African continent across Europe to above 70°N in Scandinavia and from sea level up to 3000 m a.s.l. (Conert, 1975). *A. vulneraria* is not threatened in most parts of its distribution area but has become less common in certain geographical areas (e.g. Jansen et al., 2019). The flowers of *A. vulneraria* are grouped in heads and seed mass varies between 1.9 and 4.0 mg across the studied distribution range (Daco et al., 2021). *A. vulneraria* has been found to be auto‐ or xenogamous in different populations (Couderc, 1971; Navarro, 1999). Several subspecies of *A. vulneraria* have been described as the species is very polymorphic (Cullen, 1968), but molecular genetic studies did not support the splitting into numerous subspecies (Köster et al., 2008; Nanni et al., 2004). In the present study, we did not differentiate between infraspecific taxa because we wanted to capture a large amount of genetic variation.

### 2.2 Sampling

We sampled 20 populations each of *A. vulneraria* along elevational and latitudinal gradients (Table 1). The latitudinal gradient ranged over 2000 km from the centre of the distribution of *A. vulneraria* in Central Europe to the northern distributional margin, and the elevational gradient extended from valley populations at 500 m a.s.l. to the populations at the elevational limit of the species in the Alps at 2500 m a.s.l. We defined a population as a group of plants that were at least 500 m from the next conspecific plant.

**TABLE 1:** Study sites, population size, number of samples, and genetic diversity indices for the 40 *Anthyllis vulneraria* populations sampled across elevational and latitudinal gradients.

Population	Country	Latitude (°N)	Longitude (°E)	Elevation (m a.s.l.)	*N*	*n*	*N*_G_	*N*_P_	*N*_A_	*N*_E_	*A*_R_	uH_E_	*H*_O_	*F*_IS_	*P*_HWE_
Elevational gradient															
A1	Austria	47.3980	11.2661	961	100	20	20	2	4.53	2.75	3.82	0.55	0.59	−0.06	0.306
A2	Austria	47.4421	11.6501	1521	300	20	20	1	5.65	3.01	4.46	0.57	0.56	0.03	0.282
A3	Austria	47.1606	11.7149	1810	1000	19	19	1	3.24	1.99	2.78	0.39	0.37	0.06	0.129
A4	Austria	47.1690	11.3533	1151	150	19	19	4	3.82	2.24	3.38	0.47	0.42	0.11	**0.044**
A5	Austria	47.3127	11.3894	2250	200	19	19	3	5.12	3.02	4.34	0.57	0.55	0.03	0.230
S1	Switzerland	46.1338	7.0595	545	200	19	19	2	3.94	2.63	3.55	0.53	0.51	0.04	0.121
S2	Switzerland	46.0833	7.1265	1042	80	20	20	1	3.94	2.23	3.34	0.49	0.47	0.05	0.242
S3	Switzerland	46.0496	7.9564	2162	30,000	20	20	2	4.06	2.47	3.37	0.50	0.40	0.20	**<0.001**
S4	Switzerland	46.2539	7.2734	1585	1000	20	20	0	5.53	3.24	4.50	0.63	0.58	0.08	0.063
S5	Switzerland	46.2735	7.2374	1250	1500	20	20	0	4.88	3.25	4.03	0.64	0.53	0.18	**0.004**
S6	Switzerland	46.0881	7.4067	1940	10,000	20	20	1	5.24	3.13	4.26	0.59	0.54	0.08	**0.036**
S7	Switzerland	46.1081	7.5801	2413	10,000	20	20	1	4.65	2.62	3.84	0.52	0.51	0.03	0.090
F1	France	45.0533	6.3892	2362	2000	20	20	1	3.71	2.01	2.96	0.40	0.32	0.21	**<0.001**
F2	France	45.0512	6.3533	1997	1500	17	17	1	4.24	2.59	3.71	0.52	0.39	0.26	**<0.001**
F3	France	45.1562	6.4237	1518	100	20	20	0	3.41	2.07	2.87	0.42	0.37	0.12	**0.018**
F4	France	45.2166	6.3250	1223	200	20	20	1	5.24	3.36	4.35	0.61	0.59	0.03	**0.007**
F5	France	45.0932	5.7804	471	1200	20	20	1	3.82	2.34	3.18	0.47	0.34	0.28	**<0.001**
F6	France	45.1735	6.0389	936	250	20	20	3	5.53	3.37	4.50	0.58	0.58	0.01	0.401
F7	France	45.1210	5.9852	717	200	10	10	0	3.88	2.45	3.80	0.56	0.56	0.00	0.574
F8	France	45.0598	6.3157	1807	300	19	19	0	4.29	2.68	3.64	0.50	0.46	0.07	**0.033**
Latitudinal gradient															
1	France	46.4368	4.7528	323	60	18	18	5	4.06	2.59	3.41	0.51	0.40	0.21	**<0.001**
2	France	48.1880	5.5534	443	800	19	19	1	4.06	2.67	3.54	0.52	0.46	0.11	0.610
3	Luxembourg	49.4956	5.9969	342	1000	20	20	2	4.41	2.78	3.78	0.57	0.54	0.04	0.109
4	Luxembourg	49.7314	6.2819	355	50	18	17	0	3.65	2.41	3.25	0.48	0.37	0.23	**<0.001**
5	Germany	51.2228	9.7610	442	250	20	19	1	4.00	2.69	3.48	0.54	0.47	0.15	**0.002**
6	Germany	52.0051	10.4075	191	400	20	20	0	3.71	2.37	3.23	0.48	0.47	0.03	0.949
7	Germany	54.0443	10.2290	32	1500	20	20	0	3.06	2.22	2.83	0.49	0.49	0.00	0.411
8	Germany	54.6873	9.4342	22	1500	20	20	0	3.59	2.30	3.05	0.51	0.43	0.16	**<0.001**
9	Denmark	55.5150	9.4244	42	400	20	20	0	3.88	2.41	3.32	0.50	0.52	−0.06	0.277
10	Sweden	56.3671	12.8002	81	800	15	15	0	1.94	1.30	1.77	0.18	0.15	0.15	0.189
11	Sweden	57.8892	11.9466	24	300	20	20	0	2.76	1.80	2.36	0.34	0.30	0.11	0.176
12	Sweden	58.6979	11.2199	5	100	18	18	0	3.06	1.85	2.70	0.36	0.31	0.13	0.107
13	Norway	61.0621	10.3971	438	100	20	20	1	1.88	1.44	1.76	0.23	0.18	0.21	0.716
14	Norway	62.0139	9.2074	483	75	20	9	0	1.47	1.27	1.30	0.15	0.02	0.88	**<0.001**
15	Norway	63.4409	10.6567	18	350	19	17	0	2.18	1.45	1.85	0.27	0.11	0.62	**<0.001**
16	Iceland	63.8164	−22.6970	20	10,000	20	8	0	1.41	1.32	1.38	0.17	0.00	1.00	**<0.001**
17	Norway	64.3162	12.3475	168	4000	20	12	1	1.41	1.21	1.38	0.13	0.08	0.38	**0.008**
18	Sweden	66.4261	16.8501	453	300	20	2	0	1.00	1.00	1.00	0.00	0.00	NA	—
19	Norway	67.2511	15.4282	6	1000	20	1	0	1.00	1.00	1.00	0.00	0.00	NA	—
20	Norway	68.1022	16.3783	47	1000	19	2	0	1.06	1.06	1.06	0.06	0.06	NA	—

Abbreviations: *A*_R_, allelic richness; *F*_IS_, inbreeding coefficient; *H*_O_, observed heterozygosity; *N*, population size; *n*, sample size; *N*_A_, number of alleles; *N*_E_, number of effective alleles; *N*_G_, number of multi‐locus genotype; *N*_P_, number of private alleles; *P*_HWE_, significance values of exact test for Hardy–Weinberg deviations (values in bold were significant before correction for multiple tests, values <0.03 remain significant (*p* < .05) after adjusting for the false discovery rate); uH_E_, unbiased expected heterozygosity.

During summer 2015, we collected in each population leaves from 20 plants along a 20 m long transect and put them into separate paper bags. The leaf material was preserved in silica gel until DNA extraction. The local spatial reference of each sampled individual was recorded along the transects. At each site, we also recorded the elevation above sea level and the latitude and longitude with a handheld GPS (eTrex 20, Garmin Ltd.). In small populations (<100), we recorded the number of plants to the nearest five, while in large populations we counted the plants in a part of the total area and extrapolated the number to the whole population area.

### 2.3 Genotyping using microsatellite markers

We extracted genomic DNA using a DNeasy Plant Mini Kit (QIAGEN) starting from approximately 10 mg of dried material. Samples were genotyped at 17 microsatellite loci (AV2, AV3, AV7, AV8, AV10, AV12, AV14, AV23, AV‐000290, AV‐002128, AV‐004868, AV‐005692, AV‐015354, AV‐020270, AV‐021012, AV‐021224, AV‐021803, for references see Kesselring et al., 2013 and Van Glabeke et al., 2007) in four multiplex reactions using the QIAGEN Multiplex PCR Kit (QIAGEN).

Multiplex 1 contained loci AV23 (Van Glabeke et al., 2007), AV‐000290 and AV‐015354 (Kesselring et al., 2013). Multiplex 2 contained loci AV2, AV3, AV12 (Van Glabeke et al., 2007), AV‐021012 and AV‐021224 (Kesselring et al., 2013). Multiplex 3 contained loci AV7, AV8, AV10 (Van Glabeke et al., 2007) and AV‐004868 (Kesselring et al., 2013). Multiplex 4 contained loci AV14 (Van Glabeke et al., 2007), AV‐002128, AV‐005692, AV‐020270 and AV‐021803 (Kesselring et al., 2013). We amplified each multiplex using the QIAGEN multiplex Kit (QIAGEN). Each multiplex reaction contained 1× QIAGEN multiplex master mix and 0.2 μM of each primer in a total volume of 6 μl.

The PCR conditions were: 5′ at 95°C, 30 cycles of 30″ at 95°C, 90″ at 53°C (55°C for Multiplex 1) and 30″ at 72°C and a last step of 30′ at 68°C. Reactions were performed using a Mastercycler nexus (Eppendorf). PCR products were separated using an automated sequencer (3730xl DNA Analyzer, Applied Biosystems). The data were analyzed using Geneious 11.1.5 (https://www.geneious.com, Kearse et al., 2012).

To estimate the error rate, we extracted and genotyped 5% of the samples twice. The mean error rate per sample was calculated as the number of errors divided by the total number of analyzed loci within replicated samples. We randomly chose one of the repeated samples to continue with the analyses.

### 2.4 Analysis of genetic diversity

All analyses unless otherwise stated were carried out using IBM SPSS Statistics for Windows, version 25.0 (IBM Corp.). Genetic diversity indices including number of multi‐locus genotypes (*N*_G_), number of private alleles (*N*_P_), number of alleles (*N*_A_), number of effective alleles (*N*_E_), and observed and unbiased expected heterozygosity (*H*_O_ and uH_E_, respectively) were estimated in GenAlEx 6.5 (Peakall & Smouse, 2006, 2012). Allelic richness (*A*_R_) was calculated with the R‐package PopGenKit 1.0 (Rioux Paquette, 2012) and the inbreeding coefficient (*F*_IS_, Weir & Cockerham, 1984) was calculated in FSTAT 2.9.4 (Goudet, 2003). We used regression analysis to test for the effects of elevation and latitude on diversity measures and to test for the effect of population size on uH_E_ and F_IS_. Population size was log‐transformed prior to analysis.

We tested for the significance of heterozygote deficiency or excess (Hardy–Weinberg equilibrium) in the 40 populations using the Markov chain method in GENEPOP 4.7.3 (Rousset, 2008) with 10,000 dememorisation steps, 500 batches and 10,000 subsequent iterations. The populations were tested for linkage disequilibrium among loci using an exact test based on a Markov chain method as implemented in GENEPOP. For both tests, the false discovery rate technique was used to eliminate false assignment of significance by chance (Verhoeven et al., 2005).

The software Microchecker (Van Oosterhout et al., 2004) was used to check for the presence of null alleles in each locus × population combination. Adjusted null allele frequencies were calculated with the software FreeNA (Chapuis & Estoup, 2007). The adjusted allele frequencies were used to recalculate unbiased expected heterozygosity values.

### 2.5 Analysis of population differentiation

We used *F*_ST_ (Weir & Cockerham, 1984) and *G*"_ST_ (Meirmans & Hedrick, 2011) to measure genetic differentiation among the 40 studied populations and among the populations of the elevational and latitudinal gradients separately. In addition to *F*_ST_, it has been recommended to use an alternative statistics like *G*"_ST_, which is an unbiased estimator of population differentiation suitable to infer demographic history and migration (Meirmans & Hedrick, 2011). Pairwise population *F*_ST_ and *G*"_ST_ and global *F*_ST_ and *G*"_ST_ were calculated with FSTAT 2.9.4 (Goudet, 2003) and Genodive 3.0 (Meirmans, 2020), respectively. Significance tests were based on 1000 (*F*_ST_) and 999 permutations (*G*"_ST_).

The partitioning of genetic variation within and among populations, and between the three mountain regions (France, Switzerland and Austria) was analyzed with AMOVA as implemented in GenAlEx 6.5 (Peakall & Smouse, 2006, 2012). The significance of the results was tested with 999 permutations.

Mean pairwise *G*"_ST_ values for each population were calculated by averaging all pairwise *G*"_ST_ between a population and all other populations within each gradient. They represent a measure of population divergence (Yakimowski & Eckert, 2008). We performed regression analyses to test for the effects of elevation and latitude on mean pairwise *G*"_ST_‐values. For the elevational gradient, the effects of the three Alpine regions, elevation and the interaction between region and elevation was tested in a general linear model. For the latitudinal gradient, we tested whether the relationship between pairwise *G*"_ST_ and (log) geographic distance differed between southern (1 to 9) and northern populations (10 to 20) using a permutational GLM with 5000 permutations with the R‐package lmPerm 2.1.0. (Wheeler & Torchiano, 2016). To analyze the correlation between *G*"_ST_ and uH_E_ along both gradients the effects of gradient, uH_E_ and the interaction between gradient and uH_E_ were tested in a general linear model.

We examined the relationship between genetic (pairwise *G*"_ST_/(1 − *G*"_ST_)) and geographical distances for populations of the latitudinal gradient with a Mantel test (GenAlEx 6.5; 999 permutations) to test for isolation‐by‐distance (IBD). For the populations in the Alps, we analyzed the relationship between pairwise genetic distances and both geographical distances and the differences in elevation with a linear model. *p*‐values were derived from sequential permutation tests with 1000 permutations using lmPerm. We also tested whether the mean genetic distance between pairs of populations differed between populations north of 56°N and those further south by relating the genetic distances to the geographic distances and population type using a permutational analysis of covariance with 1000 permutations using lmPerm.

### 2.6 Analysis of population genetic structure

We conducted a principal coordinate analysis (PCoA) based on pairwise *G*"_ST_‐values between populations. We fitted the two variables elevation and latitude on the ordination using the *envfit* function in the R‐package vegan 2.5‐7 (Oksanen et al., 2020).

We used STRUCTURE 2.3.4 (Pritchard et al., 2000) to analyze the genetic structure of the 40 *A. vulneraria* populations. To estimate the number of genetic clusters (*K*), we carried out ten independent runs with *K* = 1–20 with 10^6^ Markov chain Monte Carlo (MCMC) iterations after a burn‐in period of 10^5^, using the model with correlated allele frequencies and assuming admixture. We decided on the most probable number of *K* based on the log probability of the data and their variability associated with each *K* (Gilbert et al., 2012; Pritchard et al., 2007) and the consistency with the PCoA. We used CLUMPAK (Kopelman et al., 2015) to summarize the runs and generate bar plots of cluster assignments.

### 2.7 Spatial genetic structure within populations

We carried out a spatial autocorrelation analysis with SPAGeDi 1.5d (Hardy & Vekemans, 2002) using the kinship coefficient *F*_ij_ (Loiselle et al., 1995) and the local spatial coordinates of 744 individuals from 39 populations. Population A5 and five individuals from populations F3 and 10 had to be excluded from the dataset because the local coordinates had not been recorded. The intra‐population distances were divided into 10 distance classes each with a minimum of 27,637 pairs of individuals. The *F*_ij_ for each pair of individuals in each distance class was calculated and the significance levels of the means were obtained with permutation tests with 1000 permutations. Mean F_ij_ over pairs of individuals of *A. vulneraria* was plotted against mean distance for each class and the significance of the slope of that regression was obtained with a permutation test with 1000 permutations.

## 3 RESULTS

### 3.1 Genetic diversity along the elevational and latitudinal gradients

We genotyped 768 individuals with 17 polymorphic microsatellite‐markers to study the influence of the elevational and latitudinal gradients on genetic population structure and diversity. The estimated mean error rate was less than 1% per sample. We found 660 unique genotypes. Across the 40 populations, the 17 loci analyzed yielded 209 alleles, with 4–26 (mean = 12.29) alleles per locus. The number of multi‐locus genotypes (*N*_G_) was generally high but was lower in the subarctic populations and was particularly low for the three northernmost populations (no. 18, 19 and 20; Table 1). We found at least one private allele in most elevational populations, but only in six latitude populations, and those were mostly located in the southern part of the gradient (Table 1). Two of the northernmost populations (no. 18 and 19) were homozygous at every locus indicating that all individuals in the populations were fixed at the 17 microsatellite loci (*N*_A_, *N*_E_ and *A*_R_ = 1; Table 1).

**FIGURE 1:**
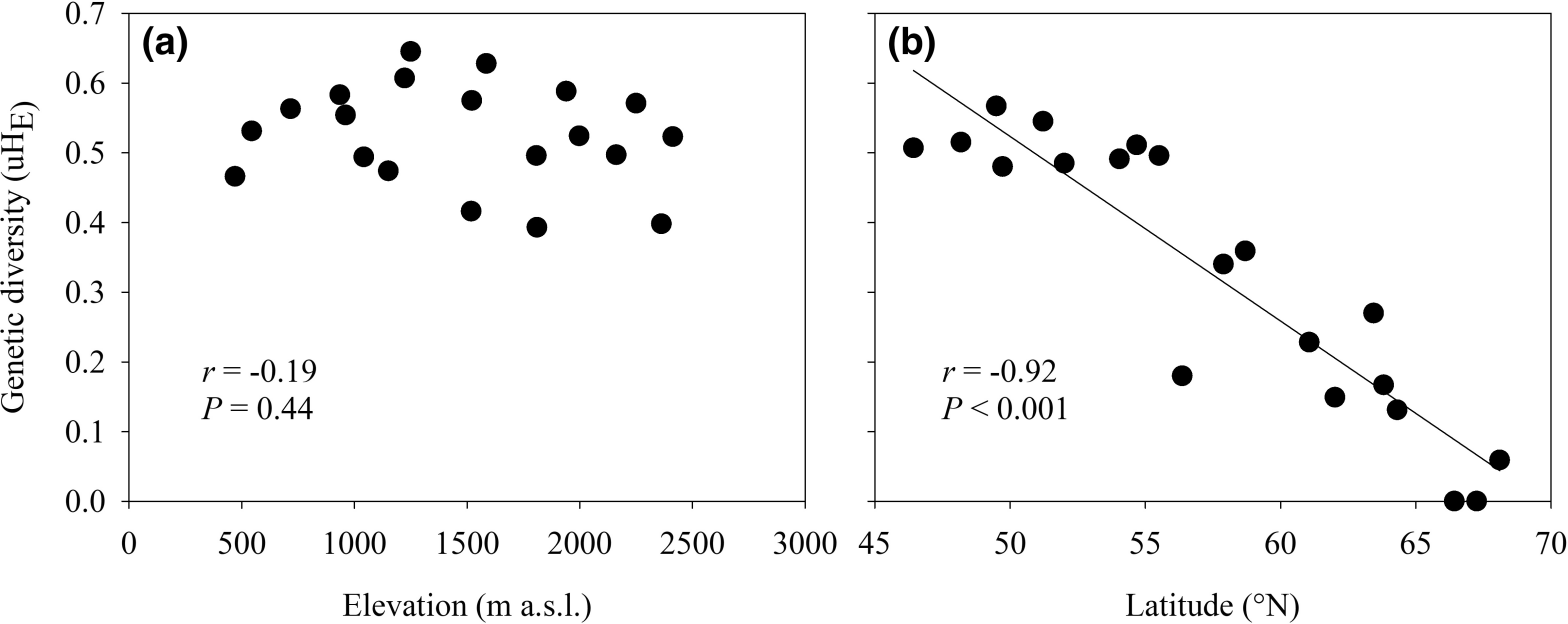
The relationship between genetic diversity and (a) elevation for the populations along the elevational gradient and (b) latitude for the populations along the latitudinal gradient.

**FIGURE 2:**
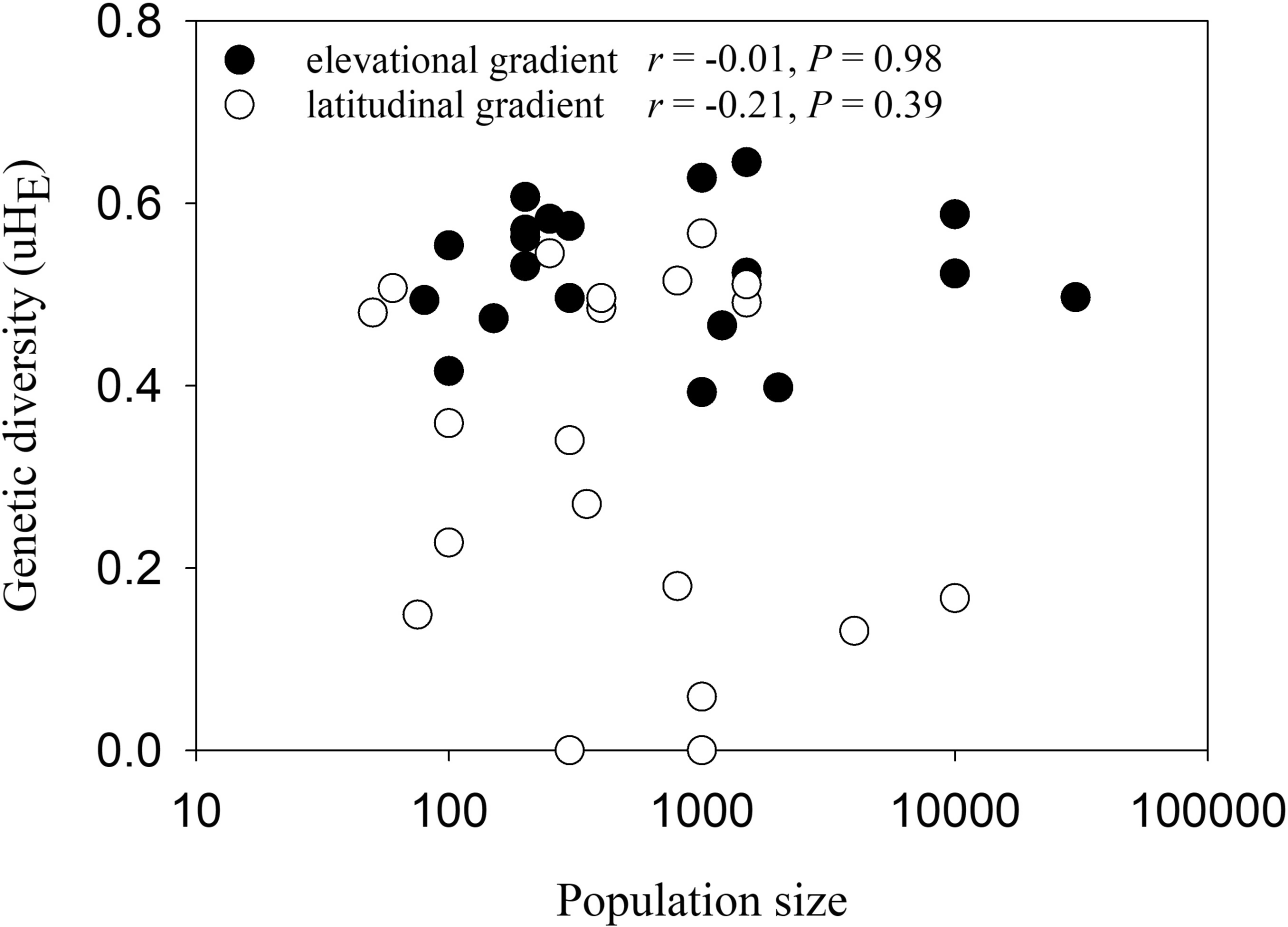
The relationship between genetic diversity (uH_E_) and size of the *A. vulneraria* populations along the elevational and latitudinal gradients.

Expected heterozygosity (uH_E_) per population varied from 0.39–0.65 (mean = 0.53) for the elevational gradient and from 0–0.57 (mean = 0.32) for the latitudinal gradient (Table 1). Genetic diversity (uH_E_) did not decrease clearly with elevation (*r* = −.19, *p* = .44; Figure 1a) and also showed no optimum (quadratic regression, *r* ^2^ = .05, *p* = .34), but decreased strongly with latitude (*r* = −.92, *p* < .001; Figure 1b). Other measures of genetic diversity also decreased with latitude (*N*_A_: *r* = −.94; *N*_E_: *r* = −.94; *A*_R_: *r* = −.94; all *p* < .001), but not with elevation (*N*_A_: *r* = .09, *p* = .72; *N*_E_: *r* = −.07, *p* = .77; *A*_R_: *r* = −.19, *p* = .94). However, closer inspection showed that genetic diversity (uH_E_) was similarly high in populations from 46 to 56°N (no. 1 to 10) and decreased strongly for the populations further north. Genetic diversity (uH_E_) did not decrease with population size (Alt.: *r* = −.01, *p* = .98; Lat.: *r* = −.21, *p* = .39) indicating that there was no strong genetic drift in small populations (Figure 2). In nearly all the populations, H_O_ was smaller than uH_E_ and ranged from 0.32 to 0.59 in the elevational and from 0 to 0.54 in the latitudinal gradient.

The inbreeding coefficient *F*_IS_ ranged from −0.06 to 0.28 in the elevational populations and from −0.06 to 1 (in populations where *H*_O_ = 0) in the populations along the latitudinal gradient (Table 1). In the most northern populations (no. 18, 19, 20), *F*_IS_ could not be calculated because nearly all the individuals of each population had identical multi‐locus genotypes leading to very small uH_E_. *F*_IS_ increased with latitude (*r* = .72, *p* < .001) but did not vary consistently with elevation (*r* = .19, *p* = .43).

We found significant deviations from Hardy–Weinberg equilibrium (HWE) for less than 10% of the allele × population combinations after correcting for the false discovery rate. Thirteen of the 17 loci significantly deviated from HWE in at least one population and 15 populations showed significant deviation from HWE (Table 1). Populations of the latitudinal gradient showed deviations from HWE from population 14 on northwards except in populations 18, 19, 20 where deviations from HWE could not be evaluated. No locus systematically deviated from HWE and no pairs of loci were systematically in linkage disequilibrium after correcting for multiple tests. All loci were therefore included in the subsequent analyses.

Null alleles were suggested in 64 locus × population combinations (9%), of which 17 were for locus AV23. However, adjusting the allele frequencies for the null alleles did not change the unbiased expected heterozygosity in any of the analyzed populations (Mann–Whitney U test, *p* > .81). Therefore, all following analyses were performed with the original data set.

### 3.2 Population genetic structure

**FIGURE 3:**
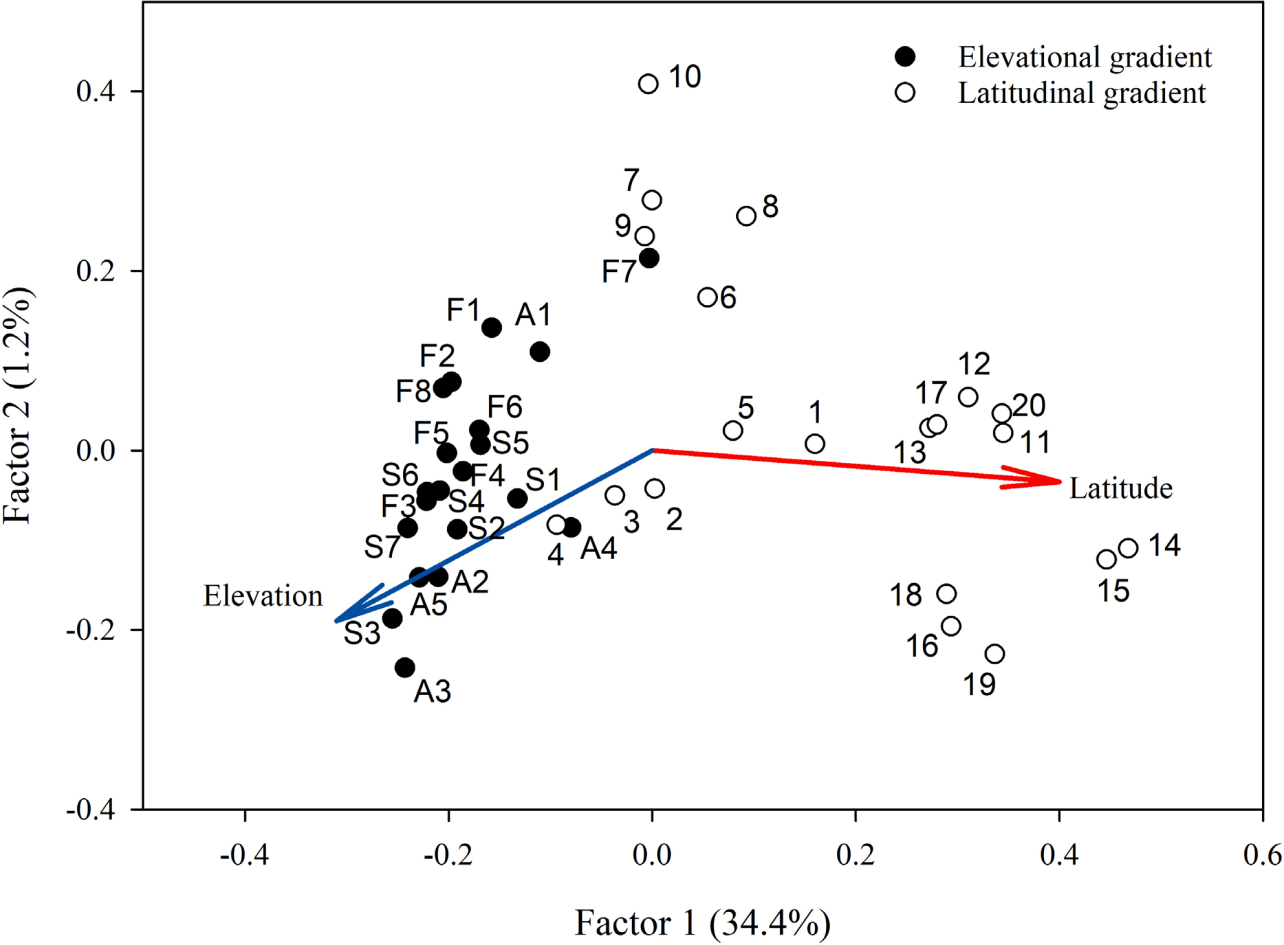
Principal coordinate analysis (PCoA; first two factors) based on pairwise *G*"_ST_‐values between all studied populations of *Anthyllis vulneraria*. The elevational and latitudinal gradients were fitted as vectors onto the ordination. Population labels correspond to abbreviations used in Table 1.

The first two axes of the PCoA explained 35.6% of the variation (Figure 3). The populations from the Alps were placed close to each other along the first PCoA axis while all population north of 56°N were placed to the right of the Central European populations. In particular, high elevation populations from the Alps and subarctic populations were widely separated from each other. Fitting of the two environmental gradients onto the ordination revealed that the genetic differentiation between populations was mainly correlated with the latitudinal gradient (*r* ^*2*^ = .81, *p* < .001 vs. *r* ^*2*^ = .65, *p* < .001 for elevation).

**FIGURE 4:**
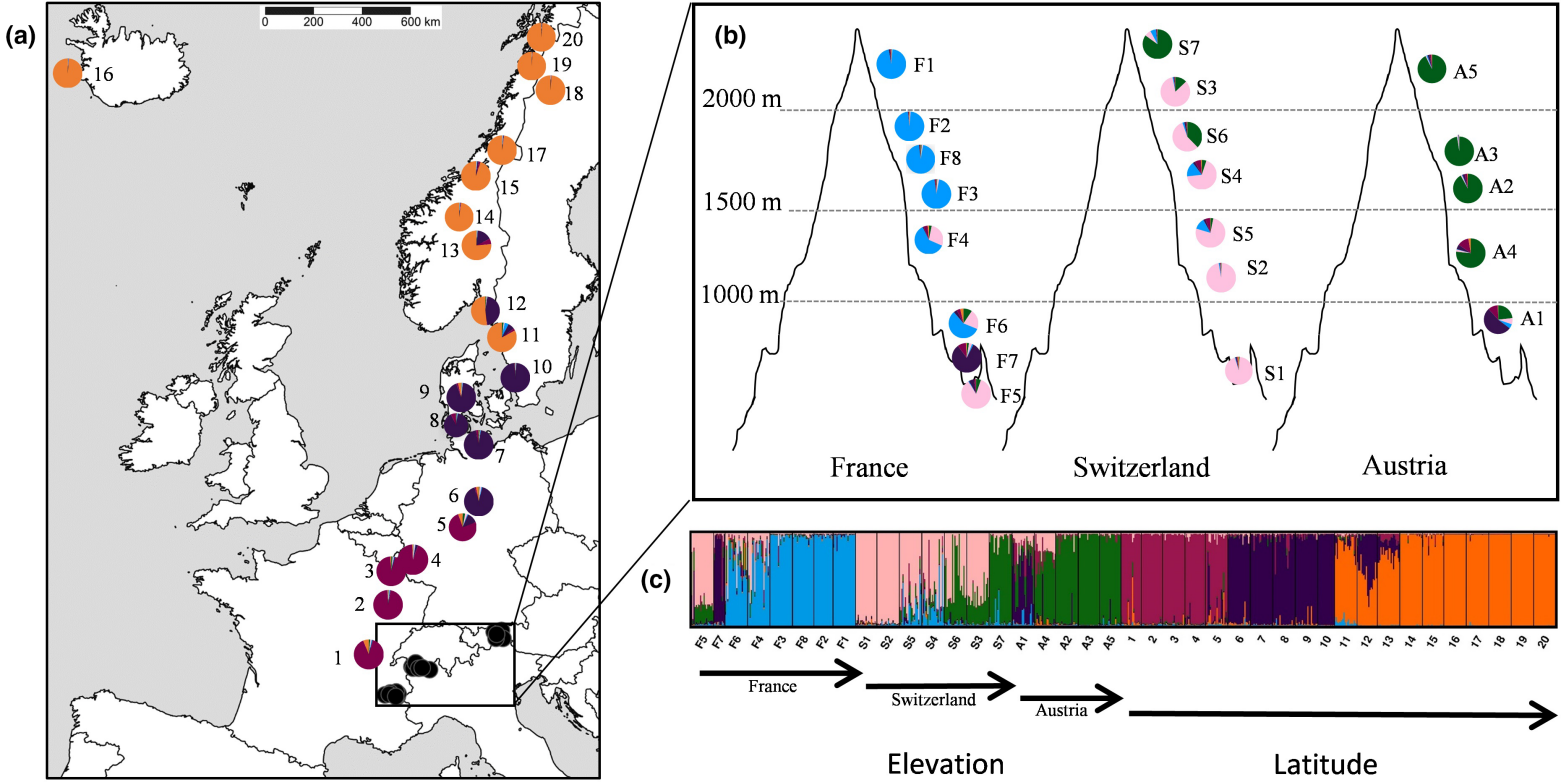
Results of the STRUCTURE analysis for 40 populations sampled across elevational and latitudinal gradients assuming *K* = 6. Pie charts in (a) and (b) represent the proportion of individuals of each population assigned to the STRUCTURE clusters. In (c), each individual is represented by a vertical line, which is partitioned into a maximum of six colored segments that represented an individual's estimated membership fractions in the six clusters. Vertical black lines separate the 40 different populations. Arrows represent increasing elevation along each of the three regions in the Alps and increasing latitude along the latitudinal gradient. For population labels see Table 1.

In the STRUCTURE analysis, the log probability of the data [ln P(D)] increased gradually and the value that also converged well across the 10 independent runs was obtained for *K* = 7 (Figure S1). However, the patterns for *K* = 6 and 7 were very similar, and we therefore preferred the lower number of groups. Structuring the populations into six clusters grouped the 10 most northern populations together (Figure 4a,b), confirming their differentiation from the Central European ones. The main difference between the patterns for *K* = 6 and 7 was a further subdivision of the northern populations (Figure S2), which was not consistent with the PCoA and probably spurious due to the very low genetic diversity of these populations. The 10 Central European populations were divided into two groups of five populations consistent with their latitudinal positions along the gradient. In the Alps, two low‐elevation populations (A1, F7) were grouped together with populations no. 6 to 10. High elevation Austrian and Swiss populations clustered together. Some admixture was present in the mid‐elevations of the three mountain regions (Figure 4b,c).

### 3.3 Population differentiation

We detected high levels of genetic differentiation among the 40 studied populations (global *F*_ST_ = 0.36 and *G*"_ST_ = 0.63). The genetic differentiation among populations was much higher across the latitudinal (*F*_ST_ = 0.46 and *G*"_ST_ = 0.69) than across the elevational gradient (*F*_ST_ = 0.19 and *G*"_ST_ = 0.40), indicating that the populations in the Alps are less differentiated from each other. AMOVA indicated that only a small proportion (4.8%) of the genetic variation was among the three mountain regions, the differentiation among populations within regions was much higher (15%). Most of the genetic variance was within populations (Table 2).

**TABLE 2:** Results of AMOVA for the populations of *Anthyllis vulneraria* along the elevational gradient with partitioning of the genetic variation between mountain regions and within and among populations.

Source of variation	df	Sum of squares	Variance components	Proportion of variation (%)	*p*‐value
Among mountain regions	2	210.37	0.27	4.79	<.001
Among populations	17	625.89	0.85	15.04	<.001
Within populations	744	3361.14	4.52	80.16	<.001

**FIGURE 5:**
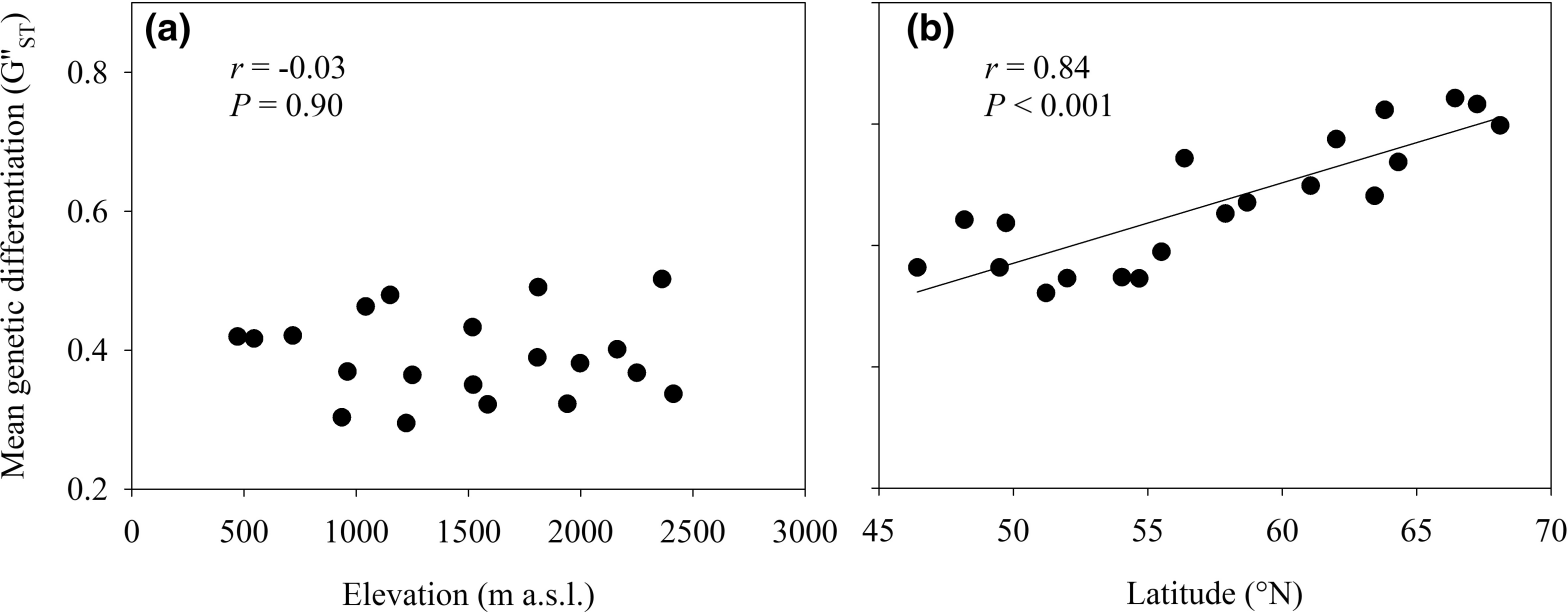
The relationship between the mean genetic distance (*G*"_ST_) of each population to all others and (a) elevation for the populations along the elevational gradient and (b) latitude for the populations along the latitudinal gradient.

**FIGURE 6:**
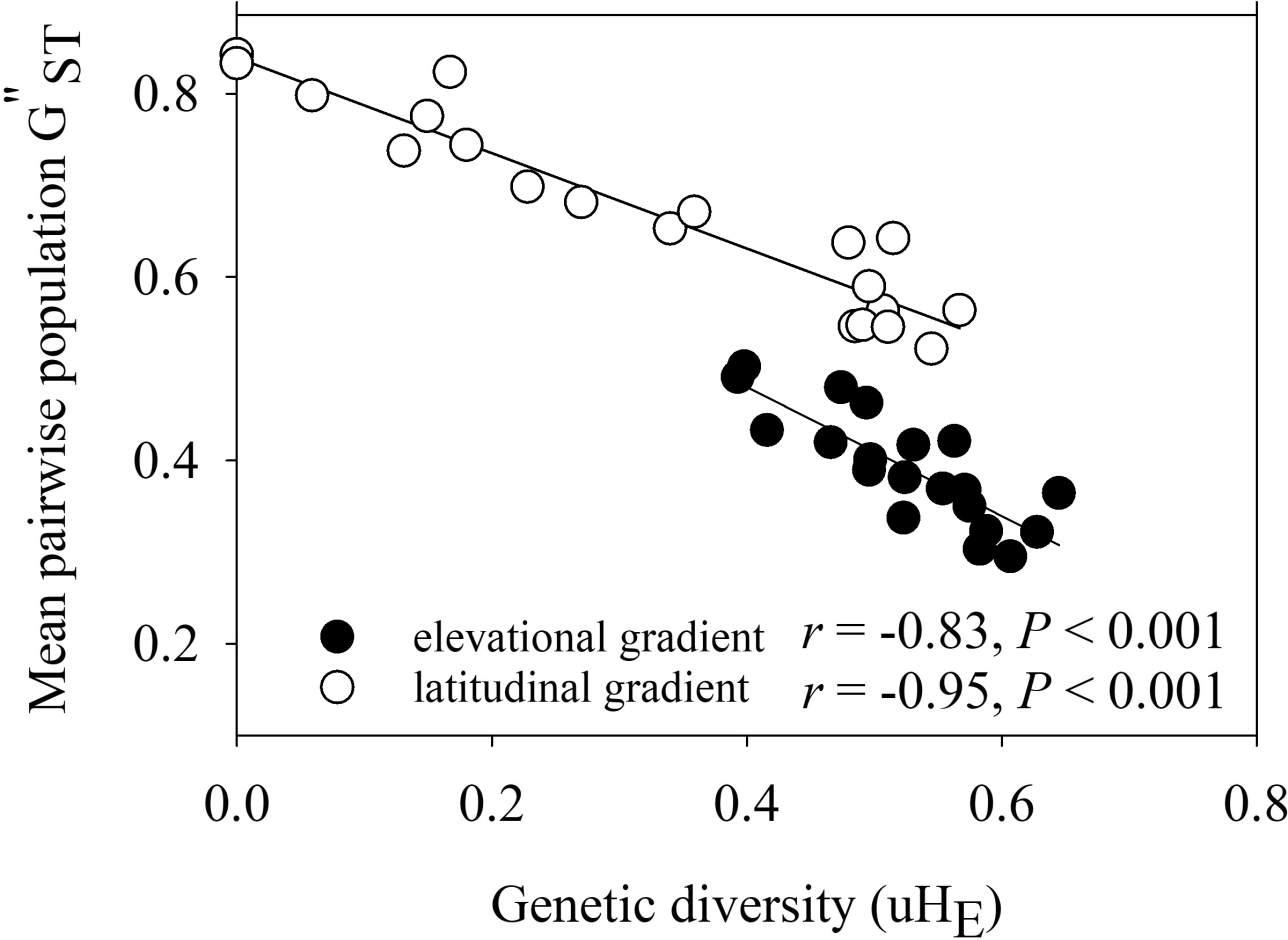
The relationship between the mean genetic distance (*G*"_ST_) of each population to all others and its genetic diversity (u*H*_E_) for populations along the elevational and latitudinal gradient.

Pairwise population *F*_ST_ and *G*"_ST_‐values between all populations are given in Table S1. Mean pairwise *G*"_ST_ of a population is a measure of genetic divergence and represents the genetic distinctness of that population from the other ones. Mean pairwise *G*"_ST_ per population within the elevational gradient did not vary with elevation (Figure 5a) and did not vary among regions (elevation: *p* = .90, region: *p* = .64, interaction: *p* = .32). In contrast, mean pairwise *G*"_ST_ increased strongly with latitude (Figure 5b) and was significantly higher for populations north of 56°N than for southern populations (*p* < .001). Mean pairwise *G*"_ST_ decreased with increasing genetic diversity (u*H*_E_) for both gradients (Figure 6), indicating absence of migration‐drift equilibrium. However, the relationships differed for the populations from the two gradients: Genetic distinctness of the latitudinal populations was much higher than that of the elevational populations (*G*"_ST_: *p* < .001), but the slope of the relationship was less steep (interaction gradient type × u*H*_E:_ *p* < .01).

**FIGURE 7:**
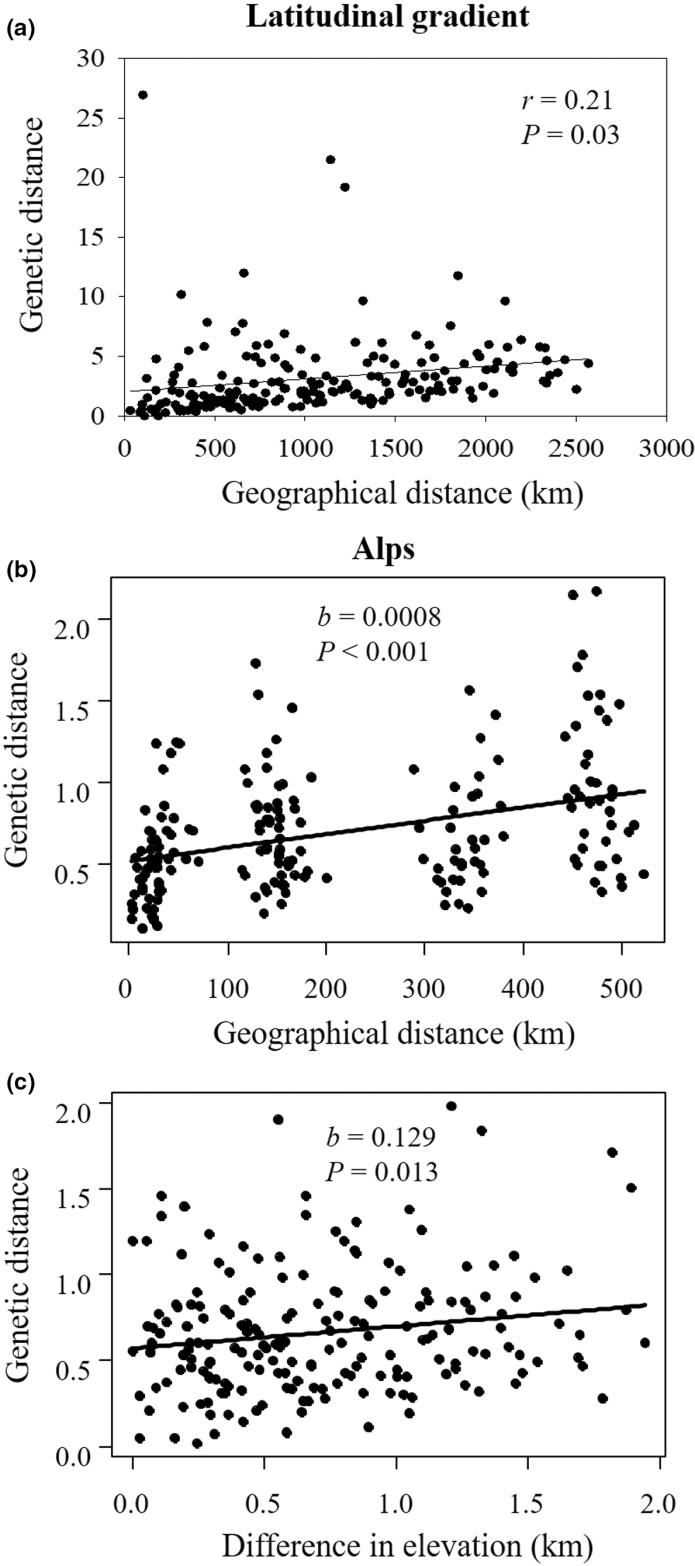
(a) Relationship between pairwise genetic distances (*G*"_ST_/(1 − *G*"_ST_)) and geographical distances between *A. vulneraria* populations along the latitudinal gradient. An outlier was removed from the analysis (see text). (b, c) partial residual plots of the relationship between pairwise genetic distances between populations in the Alps and (b) geographical distances, and (c) differences in elevation. *p*‐values are derived from Mantel tests.

Among populations of the latitudinal gradient, genetic distance *G*"_ST_/(1 − *G*"_ST_) was not related to geographical distance (*r* = .07, *p* = .25). However, after removing an outlier caused by the high mean *G*"_ST_ of northern populations, a pattern of isolation‐by‐distance (IBD) was detected for the latitudinal populations (Figure 7a, *r* = .21, *p* = .03). The mean pairwise genetic distance between populations, adjusted for the effect of geographical distance, was much higher for the northern (lat. >56°N) than for the more southern populations (5.56 ± 0.53 vs. 1.16 ± 0.71, *p* < .001).

Genetic and geographical distances of the populations in the Alps were also related (Figure 7b, *b* = 0.0008, *p* < .001). Moreover, adjusted for the effects of geographical distance, genetic differentiation between the populations in the Alps also increased with their difference in elevation (Figure 7c, *b* = 0.129, *p* = .013). The effects of 1 km difference in elevation on the genetic distance between populations were similar to those of a difference of 161.3 km in horizontal distance indicating that the effects of vertical were much stronger than those of horizontal distance. However, the maximum elevational distance between populations was only 2 km.

### 3.4 Spatial genetic structure within populations

**FIGURE 8:**
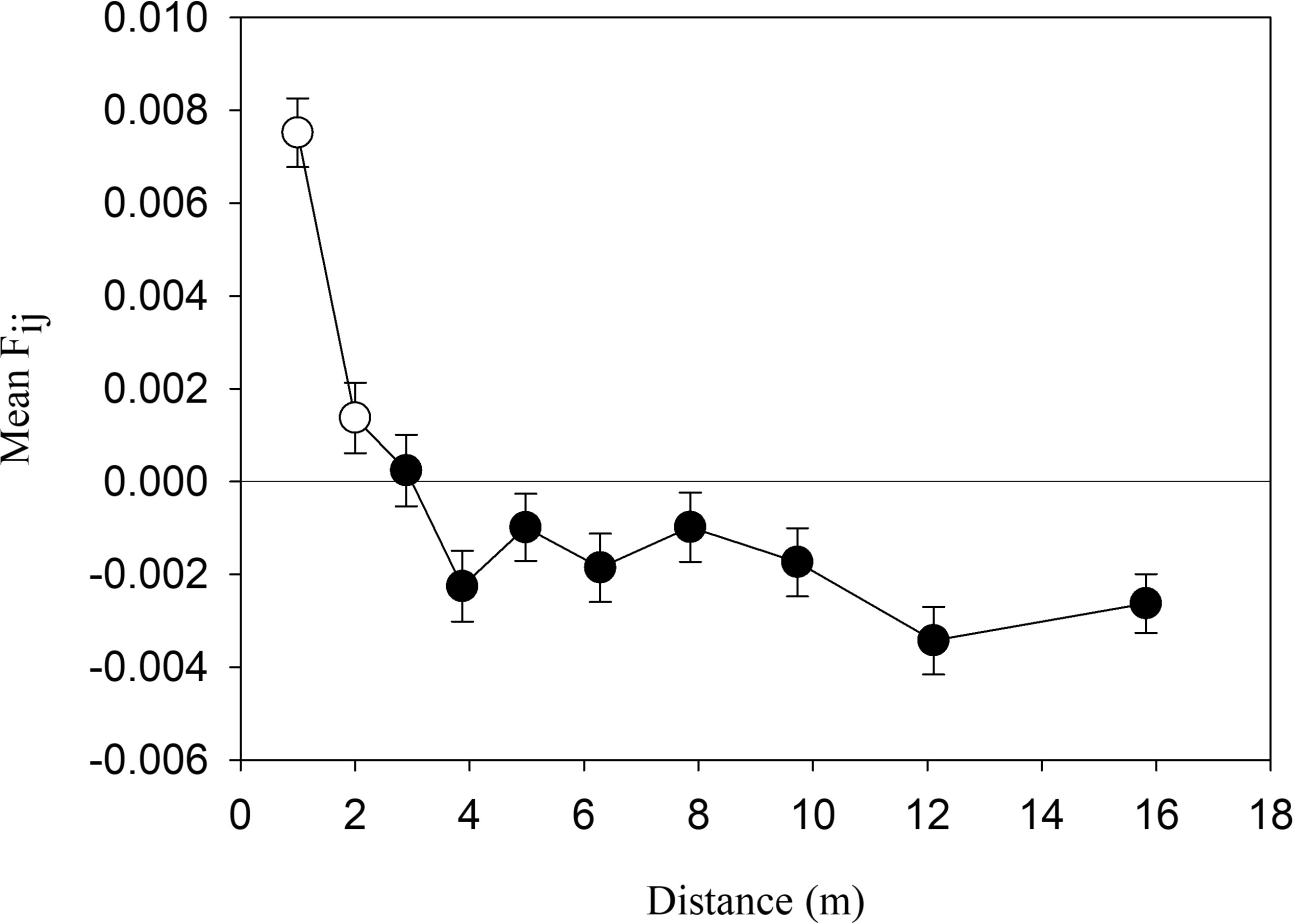
Mean kinship coefficient between pairs of individuals in 39 populations of *Anthyllis vulneraria* for 10 distance classes containing each 27,637–27,642 pairs of individuals. Means ± 1 SD. The open symbols represent significant mean kinship coefficients (*p* < .05).

Spatial autocorrelation analysis within populations showed that mean kinship coefficients decreased with distance between plants in the populations (*b* = −0.00044, *p* < .001; Figure 8). Plants growing less than 2 m from each other had a higher probability to be genetically related than plants separated by greater distances, suggesting limited gene flow due to restricted pollinator movement and limited seed dispersal.

## 4 DISCUSSION

Our results show that the patterns of genetic diversity and differentiation of the populations of the widespread plant species A*nthyllis vulneraria* differ between the elevational and latitudinal gradients. We observed a strong decline of genetic diversity with latitude down to only a single SSR‐genotype in one of the most northern populations. In contrast, no pattern of genetic diversity in relation to elevation was observed in the Alps. Similarly, in the only comparable study of both gradients, genetic diversity of populations of *Plantago lanceolata* and *P. major* also decreased with latitude but not with elevation (Halbritter et al., 2015).

The decrease of genetic variation with latitude, the much stronger genetic differentiation among northern populations and the very few private alleles present in these populations are in line with the predictions of the ACM. A decline of genetic diversity from the centre to the periphery of the distribution has been found in many species (Gougherty et al., 2020; Hirao et al., 2017; Hirsch et al., 2015; but see Casazza et al., 2021; Ilves et al., 2016; Plenk et al., 2017). However, the ACM assumes that the lower genetic diversity and stronger differentiation among peripheral populations is due to less favorable conditions, which lead to smaller and more isolated populations and subsequently to genetic erosion and strong differentiation (Eckert et al., 2008; Hardie & Hutchings, 2010; Sexton et al., 2009). In contrast, in *A. vulneraria* the size of populations increased with latitude indicating favorable conditions in the north (Daco et al., 2021), and genetic variation was not related to current population size. This suggest that not current conditions resulting in fragmentation, but historical processes (colonization after the ice age) are responsible for the much lower genetic diversity of northern populations. Other short‐lived plant species like *Arabidopsis thaliana* (Lewandowska‐Sabat et al., 2010) or *Plantago coronopus* (Berjano et al., 2015), which have migrated north after the retreat of the ice sheet after the Last Glacial Maximum also showed such genetic patterns. A similar combination of founder effects followed by demographic expansion as in *A. vulneraria* has been suggested as the reason for the population structure of Scandinavian *Trollius europaeus* (Despres et al., 2002).

The decline of genetic diversity with increasing latitude was essentially restricted to populations situated north of 56°N latitude (pop. no. 10 to 20). This limit corresponds to the southern limit of the ice‐shield during the Younger Dryas period (Stroeven et al., 2016). Populations north of this latitude also formed a distinct cluster in the STRUCTURE and PCoA analyses. The decrease of genetic diversity and the increasing differentiation with latitude suggests that northern populations lost genetic diversity due to serial founder effects during the colonization of northern Europe after the ice age, producing genetically isolated populations with very low subsequent gene flow among them (Despres et al., 2002; Excoffier et al., 2009). The rare presence of the Scandinavian cluster in lowland populations in Central Europe are in line with the hypothesis that the Scandinavian populations were founded by random individuals from lowland Central European populations that migrated north after the retreat of the ice sheets. In contrast, our results do not support for *A. vulneraria* the scenario that arctic populations of species that also occur in the Alps were founded by alpine genotypes (Albach et al., 2006; Despres et al., 2002; Ehrich et al., 2007; Schönswetter et al., 2003; Skrede et al., 2006), as no high‐elevation genotypes of the Alps were found in the Scandinavian populations of *A. vulneraria*. In contrast to studies that compared populations of arctic‐alpine species, we were able to detect the importance of the serial founder effects during recolonization after the ice‐age in *Anthyllis vulneraria* because this species has a continuous distribution from the Alps to the arctic including populations in the lowlands.

The Central European *A. vulneraria* populations were separated into a southern (no. 1–5) and a northern (no. 6–10) subgroup in the STRUCTURE and the PCoA analyses. The geographical location of this separation is reflecting another genetic signature of the last ice age as this limit corresponds to the maximum extent of continental ice sheets during the Last Glacial Maximum (LGM) some 22,000 years BP (Stroeven et al., 2016). The STRUCTURE analysis indicated a similarity between high‐elevation Swiss and Austrian populations whereas French high‐elevation populations were separated. These results suggest that after the last glacial period, glacier forelands and alpine meadows were colonized from different refugia in France than in Austria and Switzerland.

The genetic variability of peripheral populations of *A. vulneraria* at the elevational limit in the Alps was similar to that of populations at lower elevations and similar to the mean value presented in the review of Nybom (2004). This is in contrast to the predictions of the ACM that genetic erosion in small populations would lead to reduced genetic diversity in high elevation populations. However, in *A. vulneraria* the size of populations actually increased with elevation in the Alps (Daco et al., 2021) and one might thus have even expected an increase of genetic diversity with elevation, but no relationship was found. No change of genetic diversity with elevation has also been found in several other studies (Pluess & Stöcklin, 2004; see review of Hahn et al. (2012) and Ohsawa & Ide (2008)). A likely reason is gene flow among the populations along the short elevational gradient, which is supported by admixture in the mid‐elevational populations of all three regions studied.

Another non‐exclusive explanation would be that during the postglacial cold‐tolerant genotypes rapidly colonized alpine habitats in glacier forelands when the ice shields retreated due to the short geographical distances from lowland to alpine environments. The alpine environment was to those cold‐tolerant genotypes not ecologically marginal as predicted by the ACM but, in fact, corresponded largely to their ecological niche in the tundra of the lowlands. A third possible explanation would be that cold‐tolerant plants survived locally in nunataks (see Schneeweiss & Schoenswetter, 2011) and colonized in post‐glacial times glacier forelands and alpine meadows. However, in this case we would expect strong genetic differentiation among mountain regions and reduced genetic diversity in high elevation populations due to long‐term isolation and low population sizes of the source populations in the isolated nunataks (Stehlik et al., 2002). Our results are not in line with the nunatak hypothesis as genetic differentiation among mountain regions was rather low and genetic diversity did not decrease with altitude.

Along both gradients, we found significant isolation by distance patterns, indicating that gene flow is restricted and strongest between geographically close populations. However, a vertical (elevational) distance of a certain length between populations in the Alps resulted in a much stronger genetic differentiation between populations than the same horizontal distance between populations of the latitudinal gradient. This could be due to phenological differences in flowering periods that may restrict cross‐fertilization among populations at different elevations (Premoli, 2003; Reisch et al., 2005; Yamagishi et al., 2005). However, overall the genetic differentiation between valley and alpine populations of *A. vulneraria* was much smaller than between Central European and subarctic populations along the same gradient in mean annual temperature (11.5°C) because the elevational gradients were much shorter than the latitudinal gradient. The kinship analysis revealed that gene flow is even restricted over short distances within populations, which may be due to restricted pollinator movement and seed dispersal.

Peripheral populations of *A. vulneraria* in northern Europe separated by a certain spatial distance were more differentiated genetically than populations in Central Europe, indicating lower gene flow between them. A possible reason are greater mean spatial distances between neighboring populations in the North. Occurrence data from GBIF.org (2022) appear to support this, but might not be representative. A decline of population frequency toward the range periphery would be in line with the predictions of the ACM. The recent review of studies testing the ACM (Pironon et al., 2017) found that while there was only limited support for a general decline in population size, there was much stronger support for the prediction that the frequency of populations declines toward the range periphery.

## 5 CONCLUSIONS

Populations along the two gradients showed very different patterns of genetic diversity and genetic differentiation. While *A. vulneraria* maintained high amounts of genetic diversity in its Alpine and Central European populations, toward the North genetic diversity decreased strongly and genetic differentiation among populations increased due to serial founder effects during post‐glacial recolonization. Our results support the notion that postglacial latitudinal colonization over large distances results in a larger loss of genetic diversity than elevational range shifts (Ehrich et al., 2007; Hewitt, 1999). Subarctic populations differed genetically from alpine populations, indicating that the subarctic populations did not originate from the high elevational alpine ones.

The consistently high genetic diversity, allelic richness and number of private alleles across the *A. vulneraria* populations from the three Alpine regions in comparison to the Scandinavian ones indicates that the alpine populations have a higher evolutionary potential. Responses of alpine and arctic populations to climate change are thus likely to differ. The lack of genetic diversity in subarctic populations may threaten their long‐term persistence whereas in alpine populations gene flow from low‐elevation populations along the short elevational gradient could permit admixture with genotypes originating from habitats with higher temperatures.

## AUTHOR CONTRIBUTIONS

**Laura Daco:** Conceptualization (equal); data curation (supporting); formal analysis (supporting); funding acquisition (lead); investigation (lead); visualization (lead); writing – original draft (lead); writing – review and editing (equal). **Diethart Matthies:** Conceptualization (equal); investigation (equal); supervision (supporting); validation (equal); writing – review and editing (equal). **Sylvie Hermant:** Data curation (lead); formal analysis (lead). **Guy Colling:** Conceptualization (equal); investigation (equal); project administration (lead); supervision (lead); validation (equal); writing – review and editing (equal).

## CONFLICT OF INTEREST

The authors declare that they have no conflict of interest.

### 1 OPEN RESEARCH BADGES

This article has earned an Open Data badge for making publicly available the digitally‐shareable data necessary to reproduce the reported results. The data is available at https://doi.org/10.5061/dryad.ht76hdrjp.

## Supporting information


Figure S1
Figure S2
Table S1

## Data Availability

Individual genotype data are available at Dryad https://doi.org/10.5061/dryad.ht76hdrjp.
