# Supplementary material for: Genetic diversity and differentiation of populations of Anthyllis vulneraria along elevational and latitudinal gradients
Source: Ecol Evol. 2022 Aug 4;12(8):e9167. doi: 10.1002/ece3.9167 (PMC9351329; doi:10.1002/ece3.9167)
Supplement: Supplementary file 1 — Figure S1 Figure S2 Table S1 [file ECE3-12-e9167-s001.docx]

**Supporting Information for:**

**Genetic diversity and differentiation of populations of *Anthyllis vulneraria* along elevational and latitudinal gradients**

Laura Daco, Diethart Matthies, Sylvie Hermant & Guy Colling

**Fig. S1:** Inference of the number of genetic clusters *K* resulting from the STRUCTURE analysis of 40 *A. vulneraria* populations from the elevational and latitudinal gradients using a model with correlated allele frequencies and assumed admixture. Log probability of the data [ln P(D)] of ten independent runs for each number of genetic clusters inferred using STRUCTURE.


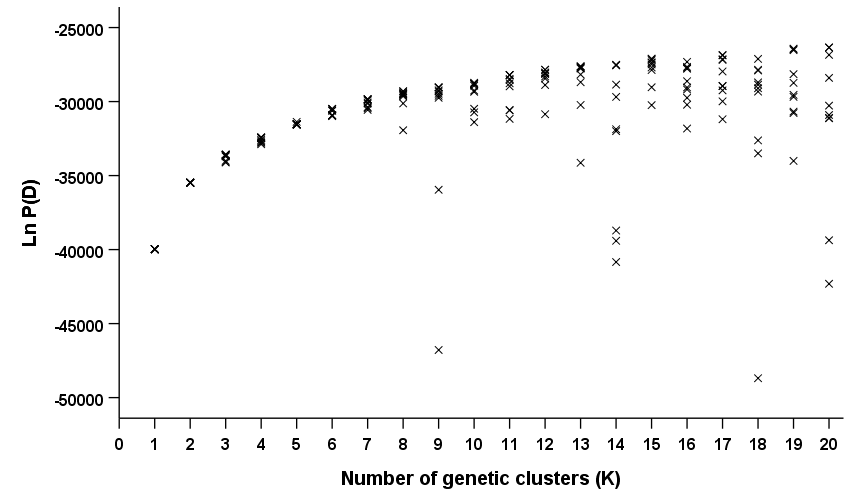

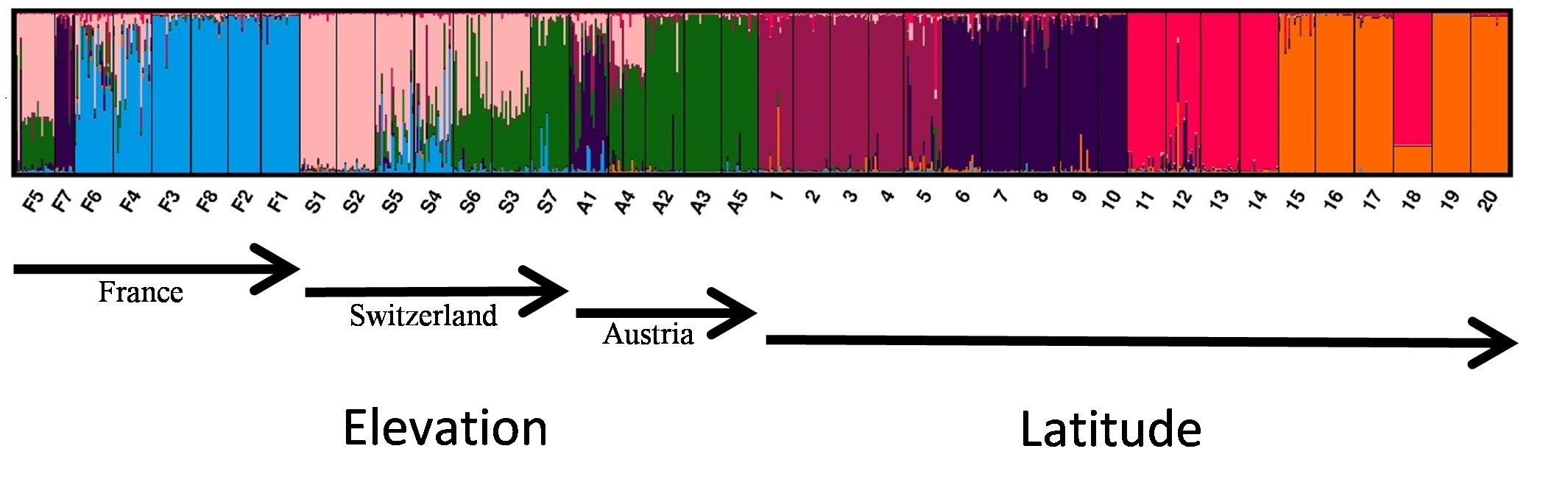


**Fig. S2:** Results of the STRUCTURE analysis for 40 populations sampled across elevational and latitudinal gradients assuming *K* = 7. Each individual is represented by a vertical line, which is partitioned into a maximum of seven coloured segments that represented an individual’s estimated membership fractions in the seven clusters. Vertical black lines separate the 40 different populations. Arrows represent increasing elevation along each of the three regions in the Alps and increasing latitude along the latitudinal gradient. For population labels see Table 1.

| **Table S1:** Pairwise F_ST_ (below diagonal) and G^′'^_ST_ (above diagonal) between populations of *Anthyllis vulneraria*. | | | | | | | | | | | | | | | | | | | | | | | | | | | | | | | | | | | | | | | | |
| --- | --- | --- | --- | --- | --- | --- | --- | --- | --- | --- | --- | --- | --- | --- | --- | --- | --- | --- | --- | --- | --- | --- | --- | --- | --- | --- | --- | --- | --- | --- | --- | --- | --- | --- | --- | --- | --- | --- | --- | --- |
|  | AAt1 | AAt2 | AAt3 | AAt4 | AAt5 | ACh1 | ACh2 | ACh3 | ACh4 | ACh5 | ACh6 | ACh7 | AFr1 | AFr2 | AFr3 | AFr4 | AFr5 | AFr6 | AFr7 | AFr8 | L1 | L2 | L3 | L4 | L5 | L6 | L7 | L8 | L9 | L10 | L11 | L12 | L13 | L14 | L15 | L16 | L17 | L18 | L19 | L20 |
| AAt1 | 0 | 0.34 | 0.55 | 0.30 | 0.31 | 0.33 | 0.46 | 0.45 | 0.35 | 0.36 | 0.31 | 0.33 | 0.51 | 0.40 | 0.46 | 0.33 | 0.34 | 0.24 | 0.23 | 0.42 | 0.46 | 0.49 | 0.32 | 0.47 | 0.30 | 0.35 | 0.27 | 0.25 | 0.33 | 0.53 | 0.62 | 0.63 | 0.67 | 0.81 | 0.74 | 0.80 | 0.70 | 0.82 | 0.83 | 0.79 |
| AAt2 | 0.15 | 0 | 0.23 | 0.40 | 0.15 | 0.42 | 0.46 | 0.25 | 0.28 | 0.38 | 0.24 | 0.22 | 0.59 | 0.45 | 0.45 | 0.31 | 0.33 | 0.27 | 0.42 | 0.47 | 0.48 | 0.50 | 0.31 | 0.40 | 0.43 | 0.47 | 0.47 | 0.56 | 0.54 | 0.69 | 0.74 | 0.71 | 0.71 | 0.85 | 0.76 | 0.82 | 0.76 | 0.85 | 0.84 | 0.85 |
| AAt3 | 0.29 | 0.12 | 0 | 0.56 | 0.27 | 0.55 | 0.59 | 0.31 | 0.50 | 0.56 | 0.35 | 0.33 | 0.68 | 0.58 | 0.60 | 0.50 | 0.46 | 0.50 | 0.61 | 0.60 | 0.67 | 0.69 | 0.57 | 0.59 | 0.59 | 0.60 | 0.63 | 0.71 | 0.71 | 0.79 | 0.83 | 0.79 | 0.86 | 0.93 | 0.86 | 0.88 | 0.83 | 0.93 | 0.90 | 0.91 |
| AAt4 | 0.15 | 0.19 | 0.31 | 0 | 0.34 | 0.40 | 0.60 | 0.53 | 0.45 | 0.48 | 0.43 | 0.33 | 0.69 | 0.58 | 0.56 | 0.46 | 0.43 | 0.45 | 0.50 | 0.63 | 0.55 | 0.59 | 0.39 | 0.56 | 0.44 | 0.48 | 0.56 | 0.49 | 0.53 | 0.76 | 0.68 | 0.70 | 0.72 | 0.82 | 0.75 | 0.85 | 0.77 | 0.85 | 0.85 | 0.79 |
| AAt5 | 0.14 | 0.06 | 0.14 | 0.16 | 0 | 0.39 | 0.50 | 0.32 | 0.29 | 0.36 | 0.18 | 0.16 | 0.63 | 0.52 | 0.49 | 0.36 | 0.36 | 0.37 | 0.43 | 0.54 | 0.55 | 0.53 | 0.37 | 0.45 | 0.48 | 0.50 | 0.51 | 0.53 | 0.52 | 0.74 | 0.73 | 0.74 | 0.75 | 0.86 | 0.80 | 0.87 | 0.78 | 0.86 | 0.89 | 0.86 |
| ACh1 | 0.15 | 0.19 | 0.30 | 0.20 | 0.18 | 0 | 0.32 | 0.39 | 0.37 | 0.42 | 0.37 | 0.41 | 0.63 | 0.46 | 0.51 | 0.31 | 0.37 | 0.36 | 0.44 | 0.45 | 0.58 | 0.55 | 0.44 | 0.45 | 0.39 | 0.44 | 0.51 | 0.48 | 0.48 | 0.70 | 0.71 | 0.66 | 0.70 | 0.83 | 0.77 | 0.82 | 0.74 | 0.84 | 0.85 | 0.78 |
| ACh2 | 0.22 | 0.21 | 0.33 | 0.31 | 0.24 | 0.16 | 0 | 0.44 | 0.33 | 0.37 | 0.43 | 0.49 | 0.65 | 0.47 | 0.52 | 0.29 | 0.50 | 0.39 | 0.51 | 0.47 | 0.61 | 0.61 | 0.49 | 0.48 | 0.48 | 0.57 | 0.57 | 0.61 | 0.59 | 0.74 | 0.79 | 0.77 | 0.78 | 0.88 | 0.81 | 0.86 | 0.79 | 0.90 | 0.87 | 0.86 |
| ACh3 | 0.21 | 0.12 | 0.17 | 0.27 | 0.15 | 0.19 | 0.22 | 0 | 0.35 | 0.43 | 0.30 | 0.34 | 0.59 | 0.46 | 0.50 | 0.30 | 0.36 | 0.34 | 0.53 | 0.45 | 0.61 | 0.56 | 0.46 | 0.40 | 0.48 | 0.54 | 0.58 | 0.64 | 0.60 | 0.74 | 0.77 | 0.74 | 0.81 | 0.89 | 0.83 | 0.83 | 0.82 | 0.89 | 0.87 | 0.86 |
| ACh4 | 0.14 | 0.11 | 0.24 | 0.20 | 0.12 | 0.16 | 0.14 | 0.15 | 0 | 0.16 | 0.21 | 0.24 | 0.47 | 0.35 | 0.35 | 0.15 | 0.39 | 0.21 | 0.35 | 0.32 | 0.52 | 0.45 | 0.31 | 0.39 | 0.44 | 0.47 | 0.45 | 0.48 | 0.41 | 0.67 | 0.68 | 0.74 | 0.68 | 0.83 | 0.75 | 0.81 | 0.76 | 0.85 | 0.85 | 0.83 |
| ACh5 | 0.14 | 0.15 | 0.27 | 0.21 | 0.14 | 0.17 | 0.16 | 0.18 | 0.06 | 0 | 0.19 | 0.29 | 0.47 | 0.36 | 0.42 | 0.22 | 0.44 | 0.28 | 0.34 | 0.37 | 0.48 | 0.46 | 0.32 | 0.47 | 0.43 | 0.48 | 0.42 | 0.44 | 0.41 | 0.67 | 0.67 | 0.67 | 0.71 | 0.83 | 0.76 | 0.80 | 0.77 | 0.83 | 0.86 | 0.83 |
| ACh6 | 0.14 | 0.10 | 0.18 | 0.20 | 0.08 | 0.16 | 0.20 | 0.14 | 0.08 | 0.07 | 0 | 0.09 | 0.54 | 0.41 | 0.45 | 0.24 | 0.35 | 0.31 | 0.31 | 0.42 | 0.54 | 0.51 | 0.35 | 0.45 | 0.45 | 0.46 | 0.40 | 0.50 | 0.39 | 0.64 | 0.73 | 0.68 | 0.72 | 0.87 | 0.77 | 0.80 | 0.78 | 0.86 | 0.85 | 0.82 |
| ACh7 | 0.15 | 0.10 | 0.18 | 0.16 | 0.07 | 0.20 | 0.24 | 0.16 | 0.10 | 0.12 | 0.04 | 0 | 0.54 | 0.41 | 0.39 | 0.29 | 0.39 | 0.30 | 0.40 | 0.44 | 0.55 | 0.57 | 0.33 | 0.46 | 0.48 | 0.47 | 0.47 | 0.53 | 0.46 | 0.69 | 0.73 | 0.72 | 0.74 | 0.88 | 0.79 | 0.84 | 0.78 | 0.87 | 0.86 | 0.84 |
| AFr1 | 0.26 | 0.30 | 0.41 | 0.39 | 0.33 | 0.34 | 0.36 | 0.32 | 0.23 | 0.23 | 0.27 | 0.29 | 0 | 0.19 | 0.38 | 0.35 | 0.59 | 0.33 | 0.48 | 0.24 | 0.69 | 0.64 | 0.58 | 0.65 | 0.58 | 0.53 | 0.52 | 0.55 | 0.55 | 0.68 | 0.77 | 0.78 | 0.80 | 0.90 | 0.84 | 0.83 | 0.79 | 0.91 | 0.88 | 0.90 |
| AFr2 | 0.18 | 0.20 | 0.32 | 0.29 | 0.23 | 0.22 | 0.23 | 0.22 | 0.15 | 0.15 | 0.18 | 0.20 | 0.10 | 0 | 0.25 | 0.21 | 0.47 | 0.16 | 0.41 | 0.10 | 0.61 | 0.56 | 0.44 | 0.52 | 0.48 | 0.48 | 0.42 | 0.51 | 0.49 | 0.65 | 0.74 | 0.69 | 0.75 | 0.87 | 0.80 | 0.83 | 0.74 | 0.88 | 0.85 | 0.85 |
| AFr3 | 0.24 | 0.23 | 0.36 | 0.31 | 0.25 | 0.27 | 0.28 | 0.27 | 0.17 | 0.20 | 0.23 | 0.20 | 0.23 | 0.13 | 0 | 0.27 | 0.56 | 0.25 | 0.53 | 0.27 | 0.64 | 0.60 | 0.46 | 0.53 | 0.55 | 0.58 | 0.57 | 0.62 | 0.56 | 0.77 | 0.77 | 0.81 | 0.78 | 0.90 | 0.82 | 0.82 | 0.78 | 0.89 | 0.88 | 0.87 |
| AFr4 | 0.14 | 0.13 | 0.25 | 0.21 | 0.15 | 0.13 | 0.13 | 0.13 | 0.06 | 0.08 | 0.10 | 0.13 | 0.18 | 0.09 | 0.13 | 0 | 0.37 | 0.13 | 0.32 | 0.19 | 0.47 | 0.42 | 0.32 | 0.35 | 0.34 | 0.43 | 0.42 | 0.46 | 0.41 | 0.63 | 0.68 | 0.71 | 0.65 | 0.83 | 0.72 | 0.76 | 0.74 | 0.82 | 0.81 | 0.79 |
| AFr5 | 0.17 | 0.16 | 0.26 | 0.23 | 0.17 | 0.19 | 0.26 | 0.19 | 0.18 | 0.19 | 0.17 | 0.19 | 0.33 | 0.24 | 0.31 | 0.17 | 0 | 0.37 | 0.44 | 0.44 | 0.57 | 0.61 | 0.50 | 0.51 | 0.48 | 0.40 | 0.47 | 0.50 | 0.49 | 0.65 | 0.76 | 0.70 | 0.78 | 0.88 | 0.80 | 0.86 | 0.79 | 0.90 | 0.88 | 0.84 |
| AFr6 | 0.10 | 0.11 | 0.25 | 0.21 | 0.16 | 0.16 | 0.18 | 0.15 | 0.08 | 0.11 | 0.13 | 0.14 | 0.17 | 0.07 | 0.13 | 0.05 | 0.17 | 0 | 0.33 | 0.17 | 0.47 | 0.44 | 0.31 | 0.39 | 0.35 | 0.41 | 0.36 | 0.42 | 0.42 | 0.59 | 0.65 | 0.66 | 0.70 | 0.81 | 0.71 | 0.78 | 0.71 | 0.82 | 0.83 | 0.79 |
| AFr7 | 0.10 | 0.18 | 0.33 | 0.24 | 0.19 | 0.20 | 0.25 | 0.25 | 0.14 | 0.13 | 0.13 | 0.18 | 0.26 | 0.19 | 0.28 | 0.13 | 0.22 | 0.14 | 0 | 0.42 | 0.38 | 0.43 | 0.32 | 0.43 | 0.32 | 0.17 | 0.01 | 0.16 | 0.14 | 0.37 | 0.58 | 0.58 | 0.60 | 0.79 | 0.63 | 0.75 | 0.62 | 0.81 | 0.78 | 0.71 |
| AFr8 | 0.20 | 0.22 | 0.33 | 0.32 | 0.25 | 0.22 | 0.24 | 0.22 | 0.14 | 0.16 | 0.19 | 0.22 | 0.13 | 0.05 | 0.15 | 0.09 | 0.23 | 0.08 | 0.20 | 0 | 0.63 | 0.58 | 0.49 | 0.54 | 0.51 | 0.50 | 0.46 | 0.52 | 0.48 | 0.66 | 0.74 | 0.76 | 0.75 | 0.88 | 0.81 | 0.82 | 0.74 | 0.89 | 0.85 | 0.86 |
| L1 | 0.22 | 0.22 | 0.37 | 0.28 | 0.25 | 0.28 | 0.30 | 0.30 | 0.22 | 0.20 | 0.24 | 0.26 | 0.38 | 0.29 | 0.34 | 0.21 | 0.29 | 0.21 | 0.18 | 0.31 | 0 | 0.50 | 0.32 | 0.44 | 0.33 | 0.43 | 0.43 | 0.44 | 0.52 | 0.70 | 0.49 | 0.56 | 0.60 | 0.67 | 0.60 | 0.81 | 0.65 | 0.73 | 0.78 | 0.69 |
| L2 | 0.23 | 0.23 | 0.38 | 0.30 | 0.24 | 0.26 | 0.30 | 0.28 | 0.19 | 0.19 | 0.23 | 0.27 | 0.35 | 0.27 | 0.32 | 0.18 | 0.31 | 0.20 | 0.20 | 0.29 | 0.25 | 0 | 0.36 | 0.38 | 0.42 | 0.57 | 0.51 | 0.52 | 0.59 | 0.47 | 0.38 | 0.41 | 0.42 | 0.52 | 0.41 | 0.54 | 0.51 | 0.59 | 0.65 | 0.85 |
| L3 | 0.14 | 0.13 | 0.29 | 0.19 | 0.16 | 0.20 | 0.23 | 0.22 | 0.12 | 0.13 | 0.15 | 0.15 | 0.30 | 0.20 | 0.23 | 0.13 | 0.24 | 0.13 | 0.14 | 0.23 | 0.15 | 0.16 | 0 | 0.31 | 0.30 | 0.46 | 0.39 | 0.43 | 0.45 | 0.42 | 0.31 | 0.34 | 0.34 | 0.49 | 0.38 | 0.52 | 0.44 | 0.50 | 0.57 | 0.58 |
| L4 | 0.23 | 0.19 | 0.33 | 0.29 | 0.21 | 0.22 | 0.24 | 0.20 | 0.17 | 0.20 | 0.21 | 0.23 | 0.36 | 0.26 | 0.29 | 0.16 | 0.26 | 0.18 | 0.21 | 0.28 | 0.22 | 0.19 | 0.15 | 0 | 0.36 | 0.50 | 0.51 | 0.56 | 0.54 | 0.48 | 0.38 | 0.41 | 0.42 | 0.58 | 0.47 | 0.58 | 0.53 | 0.64 | 0.66 | 0.65 |
| L5 | 0.14 | 0.19 | 0.31 | 0.21 | 0.21 | 0.18 | 0.23 | 0.23 | 0.18 | 0.18 | 0.20 | 0.23 | 0.31 | 0.22 | 0.28 | 0.14 | 0.23 | 0.15 | 0.14 | 0.24 | 0.16 | 0.20 | 0.13 | 0.18 | 0 | 0.35 | 0.35 | 0.30 | 0.43 | 0.38 | 0.28 | 0.30 | 0.33 | 0.45 | 0.35 | 0.50 | 0.44 | 0.52 | 0.54 | 0.52 |
| L6 | 0.17 | 0.22 | 0.33 | 0.25 | 0.24 | 0.22 | 0.29 | 0.28 | 0.21 | 0.21 | 0.21 | 0.23 | 0.30 | 0.24 | 0.32 | 0.19 | 0.21 | 0.19 | 0.08 | 0.26 | 0.22 | 0.29 | 0.22 | 0.26 | 0.17 | 0 | 0.21 | 0.29 | 0.26 | 0.33 | 0.35 | 0.32 | 0.42 | 0.53 | 0.39 | 0.50 | 0.40 | 0.61 | 0.58 | 0.53 |
| L7 | 0.13 | 0.22 | 0.35 | 0.29 | 0.24 | 0.25 | 0.29 | 0.29 | 0.20 | 0.18 | 0.18 | 0.23 | 0.29 | 0.21 | 0.31 | 0.19 | 0.24 | 0.17 | 0.00 | 0.23 | 0.21 | 0.25 | 0.19 | 0.26 | 0.17 | 0.11 | 0 | 0.23 | 0.20 | 0.21 | 0.37 | 0.33 | 0.42 | 0.55 | 0.41 | 0.54 | 0.47 | 0.64 | 0.61 | 0.56 |
| L8 | 0.12 | 0.26 | 0.39 | 0.25 | 0.24 | 0.23 | 0.30 | 0.32 | 0.21 | 0.18 | 0.22 | 0.26 | 0.30 | 0.24 | 0.33 | 0.20 | 0.25 | 0.19 | 0.08 | 0.26 | 0.21 | 0.25 | 0.20 | 0.28 | 0.14 | 0.14 | 0.12 | 0 | 0.28 | 0.30 | 0.29 | 0.30 | 0.38 | 0.49 | 0.41 | 0.54 | 0.45 | 0.56 | 0.62 | 0.56 |
| L9 | 0.16 | 0.25 | 0.39 | 0.27 | 0.24 | 0.24 | 0.30 | 0.30 | 0.18 | 0.17 | 0.18 | 0.23 | 0.31 | 0.24 | 0.30 | 0.19 | 0.25 | 0.20 | 0.07 | 0.24 | 0.26 | 0.29 | 0.21 | 0.27 | 0.21 | 0.13 | 0.10 | 0.14 | 0 | 0.34 | 0.38 | 0.37 | 0.43 | 0.56 | 0.43 | 0.53 | 0.49 | 0.64 | 0.61 | 0.57 |
| L10 | 0.32 | 0.41 | 0.55 | 0.50 | 0.45 | 0.44 | 0.47 | 0.47 | 0.38 | 0.37 | 0.37 | 0.43 | 0.47 | 0.41 | 0.53 | 0.37 | 0.42 | 0.35 | 0.26 | 0.42 | 0.45 | 0.74 | 0.70 | 0.74 | 0.63 | 0.52 | 0.34 | 0.48 | 0.54 | 0 | 0.83 | 0.77 | 0.77 | 0.92 | 0.86 | 0.91 | 0.87 | 0.96 | 0.95 | 0.91 |
| L11 | 0.34 | 0.40 | 0.52 | 0.40 | 0.40 | 0.40 | 0.46 | 0.45 | 0.35 | 0.34 | 0.39 | 0.41 | 0.49 | 0.42 | 0.48 | 0.36 | 0.45 | 0.35 | 0.34 | 0.43 | 0.28 | 0.67 | 0.57 | 0.64 | 0.51 | 0.60 | 0.63 | 0.50 | 0.66 | 0.60 | 0 | 0.49 | 0.63 | 0.60 | 0.67 | 0.83 | 0.73 | 0.78 | 0.83 | 0.75 |
| L12 | 0.34 | 0.38 | 0.49 | 0.41 | 0.39 | 0.37 | 0.44 | 0.42 | 0.37 | 0.33 | 0.36 | 0.40 | 0.48 | 0.39 | 0.50 | 0.37 | 0.41 | 0.35 | 0.33 | 0.43 | 0.32 | 0.73 | 0.64 | 0.71 | 0.54 | 0.55 | 0.57 | 0.53 | 0.65 | 0.56 | 0.32 | 0 | 0.74 | 0.62 | 0.70 | 0.81 | 0.74 | 0.80 | 0.85 | 0.73 |
| L13 | 0.41 | 0.43 | 0.59 | 0.47 | 0.46 | 0.44 | 0.50 | 0.52 | 0.39 | 0.40 | 0.43 | 0.46 | 0.55 | 0.47 | 0.53 | 0.38 | 0.50 | 0.41 | 0.40 | 0.48 | 0.38 | 0.67 | 0.56 | 0.65 | 0.53 | 0.65 | 0.66 | 0.61 | 0.67 | 0.61 | 0.45 | 0.53 | 0 | 0.76 | 0.68 | 0.83 | 0.73 | 0.83 | 0.85 | 0.83 |
| L14 | 0.53 | 0.54 | 0.68 | 0.57 | 0.56 | 0.55 | 0.59 | 0.60 | 0.51 | 0.49 | 0.55 | 0.58 | 0.66 | 0.59 | 0.65 | 0.51 | 0.61 | 0.51 | 0.56 | 0.60 | 0.45 | 0.78 | 0.77 | 0.83 | 0.69 | 0.77 | 0.81 | 0.74 | 0.83 | 0.78 | 0.46 | 0.47 | 0.62 | 0 | 0.68 | 0.87 | 0.80 | 0.88 | 0.89 | 0.82 |
| L15 | 0.43 | 0.44 | 0.58 | 0.47 | 0.46 | 0.46 | 0.50 | 0.51 | 0.41 | 0.41 | 0.43 | 0.48 | 0.56 | 0.49 | 0.54 | 0.40 | 0.51 | 0.41 | 0.39 | 0.50 | 0.36 | 0.68 | 0.66 | 0.75 | 0.60 | 0.64 | 0.67 | 0.68 | 0.70 | 0.66 | 0.47 | 0.48 | 0.51 | 0.54 | 0 | 0.69 | 0.60 | 0.85 | 0.59 | 0.66 |
| L16 | 0.51 | 0.51 | 0.64 | 0.58 | 0.55 | 0.53 | 0.57 | 0.55 | 0.49 | 0.47 | 0.49 | 0.55 | 0.59 | 0.55 | 0.58 | 0.47 | 0.59 | 0.48 | 0.53 | 0.55 | 0.54 | 0.82 | 0.82 | 0.85 | 0.77 | 0.75 | 0.81 | 0.82 | 0.79 | 0.75 | 0.62 | 0.60 | 0.66 | 0.74 | 0.54 | 0 | 0.86 | 0.92 | 0.79 | 0.88 |
| L17 | 0.46 | 0.50 | 0.62 | 0.54 | 0.53 | 0.50 | 0.55 | 0.57 | 0.47 | 0.48 | 0.51 | 0.53 | 0.58 | 0.52 | 0.58 | 0.49 | 0.56 | 0.47 | 0.48 | 0.51 | 0.45 | 0.75 | 0.68 | 0.77 | 0.64 | 0.56 | 0.67 | 0.63 | 0.68 | 0.74 | 0.53 | 0.58 | 0.54 | 0.70 | 0.48 | 0.69 | 0 | 0.91 | 0.85 | 0.89 |
| L18 | 0.60 | 0.60 | 0.75 | 0.66 | 0.62 | 0.62 | 0.67 | 0.66 | 0.58 | 0.56 | 0.60 | 0.63 | 0.73 | 0.67 | 0.71 | 0.57 | 0.69 | 0.58 | 0.67 | 0.68 | 0.55 | 0.80 | 0.71 | 0.84 | 0.72 | 0.82 | 0.86 | 0.77 | 0.86 | 0.89 | 0.64 | 0.66 | 0.74 | 0.81 | 0.74 | 0.84 | 0.83 | 0 | 1.00 | 0.98 |
| L19 | 0.60 | 0.61 | 0.73 | 0.66 | 0.64 | 0.63 | 0.66 | 0.65 | 0.58 | 0.58 | 0.60 | 0.64 | 0.71 | 0.65 | 0.70 | 0.57 | 0.67 | 0.59 | 0.67 | 0.65 | 0.60 | 0.86 | 0.80 | 0.86 | 0.74 | 0.77 | 0.81 | 0.83 | 0.82 | 0.88 | 0.69 | 0.71 | 0.76 | 0.83 | 0.52 | 0.72 | 0.74 | 1.00 | 0 | 0.96 |
| L20 | 0.56 | 0.61 | 0.73 | 0.59 | 0.63 | 0.57 | 0.66 | 0.65 | 0.58 | 0.57 | 0.58 | 0.63 | 0.72 | 0.64 | 0.70 | 0.57 | 0.63 | 0.56 | 0.63 | 0.64 | 0.55 | 0.64 | 0.78 | 0.85 | 0.68 | 0.69 | 0.74 | 0.73 | 0.74 | 0.85 | 0.61 | 0.63 | 0.71 | 0.80 | 0.63 | 0.80 | 0.82 | 1.00 | 1.00 | 0 |
